# Evaluation of the Antibacterial and Antioxidant Activity of *Mimosa rubicaulis* and *Reinwardtia indica*

**DOI:** 10.1155/2020/3862642

**Published:** 2020-11-10

**Authors:** Roshani Gurung, Sundar Adhikari, Kalpana Parajuli

**Affiliations:** Department of Pharmaceutical Sciences, School of Health and Allied Sciences, Pokhara University, Pokhara-30, Kaski 33700, Nepal

## Abstract

Many plants have the property of wound healing, but most of the people are using costly allopathic medicine for the wound. This might be due to lack of awareness about the traditional uses and lack of scientific study of ethnomedicinal plants. So, this study aimed to carry out the antibacterial and antioxidant activity of two medicinal plants which are used traditionally for wound healing activity, i.e.,*Mimosa rubicaulis* and *Reinwardtia indica.* Different parts of *Mimosa rubicaulis* (root, stem, and leaves) and *Reinwardtia indica* (flower and leaves) were used for the study. Extractions were done by maceration using ethanol as solvent. Antibacterial activity was carried out by the well diffusion method, and antioxidant activities were screened by DPPH radical scavenging and NO scavenging assays. Extract of *M. rubicaulis* has shown a weak zone of inhibition towards *S. aureus* and *P. vulgaris* whereas *R. indica* has shown no zone of inhibition towards selected bacterial strains. Leaf, root, and stem extracts of *M. rubicaulis* have shown potent antioxidant activity, i.e., IC_50_ value of 9.8 *µ*g/ml, 10.19 *µ*g/ml, and, 13.64 *µ*g/ml, respectively. Similarly, leaf extract of *R. indica* exhibited antioxidant activity with an IC_50_ value of 19.73 *μ*g/ml. The percentage inhibition of NO radicals of root and stem of *M. rubicaulis* and leaves of *R. indica* was 31%, 21%, and 22%, respectively. Out of these two plant species, *M. rubicaulis* had shown antibacterial activity towards selected microorganism, but antioxidant activity was shown by both plant species. These properties on above mentioned two plant species might help for the development of a marketed formulation for antibacterial and wound-healing agent since wound healing is promoted by antibacterial and antioxidant activities.

## 1. Introduction

In developing countries, infectious diseases due to bacteria are the main causes of morbidity and mortality among the general population [1]. In the present context, various antibiotics have been discovered for the treatment of infectious diseases. It was assumed that infectious diseases will no longer exist after the discovery of antibiotics [2]. However, the increased use of antibiotics has led to the drug-resistant strain of bacteria which in turn has resulted in the emergence of new infectious diseases. So, it is a necessity and challenge to develop new antibiotics with novel mechanisms of action to overcome the problem of antibiotic resistance [3].

Today's another issue is that many people in this fast-growing world are suffering from diseases such as heart disease, cancer, Alzheimer's disease, neurological disorders, hypertension, diabetes mellitus, renal failure, liver diseases, and early aging [4] due to exposure on various exogenous sources such as radiations, various toxic chemicals, and pollution [5]. That might be due to the generation of free radicals [6] which are capable of attacking the healthy cells of the body, causing them to lose their structure and function [7].

An antioxidant is a molecule that inhibits the oxidation of other molecules and terminates the chain reactions of free radicals by donating an electron or hydrogen atom to free radical causing the stable chemicals [8]. Free radicals can be scavenged by the in vivo production of antioxidant compounds, but the endogenous antioxidants are insufficient to remove them completely and maintain a balance. As a result, dietary antioxidants are required to counteract excess free radicals [9].

Different phytochemicals are present in plants such as terpenoids, aromatic and aliphatic compounds, carbohydrates, lactams, peptides, and quinines where these chemicals are responsible for antimicrobial activity of some plants [2]. Some plants have natural antioxidant activity as they may contain vitamin C, vitamin E, carotenes, flavonoids, isoflavones, anthocyanin, catechin, and isocatechin [10]. Due to the presence of such phytochemicals, plants are widely used as medicine ethnomedicinally. The primary benefits of using plant-derived medicines are relatively safer with fewer side effects than synthetic alternatives, offering profound therapeutic benefits and more affordable treatments [11].

Although plants have traditional uses as medicine, which are safer for use, most of the people use allopathic medicine for their primary healthcare. Also, traditionally used plants have not been extensively studied scientifically yet. So, the main aim of this study was to determine the antibacterial and antioxidant activity of *Mimosa rubicaulis* and *Reinwardtia indica* which are used for wound-healing activity traditionally.

*Mimosa rubicaulis* belongs to the Leguminosae family. It is a straggling shrub. Traditionally, it is used in peptic ulcer, dislocated bone, sprains, backache, hemorrhoids, wound, and fever [12]. *Reinwardtia indica* belongs to the Linaceae family. It is an erect perennial herb about 1 m high. Traditionally, plant is applied to boils, headaches, scabies, wounds, bee stings, insect's bites, and thorn stabs [12].

## 2. Materials and Methods

### 2.1. Plant Material

Different parts of plant samples, i.e., root, stem, and leaves of *M. rubicaulis*, and flower and leaves of *R. indica* were collected from Pokhara Valley, Nepal. Proper identification of plants was done under the supervision of local traditional healers. The collected samples of crude drugs were properly identified by the botanist Prof. Dr. Radhe Shyam Kayastha and preserved in the Pharmacognosy Laboratory of School of Health and Allied Sciences, Pokhara University, Nepal.

### 2.2. Chemicals

Ascorbic acid (Loba Chemie), dimethyl sulphoxide (Alpha Chemika), 2,2-diphenyl-1-picrylhydrazyl (DPPH) (Wako Pure Chemical), methanol (Fischer Scientific), Muller Hilton Agar (Himedia Laboratories), N-(1-naphthyl)ethylenediamine (NEDD) (Loba Chemie), sodium nitroprusside (Fischer Scientific), and all the other required chemicals were obtained from various chemical suppliers as well from the Laboratory of School of Health and Allied Sciences, Pokhara University, Pokhara-30, Kaski, Nepal.

### 2.3. Extraction

Shade-dried samples were extracted by a double maceration process using ethanol as solvent. For this, 25 g of each sample was weighed and macerated in 175 ml ethanol (ethanol: crude drug = 7 : 1) for 24 hr. After 24 hr, filtration was done by using filter paper and the obtained residue was again macerated with 175 ml, i.e., the same volume of ethanol for 24 hr. Then, obtained filtrates were mixed and solvent evaporation was done by using a rotatory vacuum evaporator and collected in the Petri dish. Again, the samples were kept in a vacuum desiccator for the complete removal of the solvent present in the extract. The obtained dried extracts were collected in a sample vial. They were labeled with symbols using alphabetical letters and preserved in the refrigerator.

## 3. Antibacterial Activity

### 3.1. Bacterial Strain and Its Growth Condition

The bacterial strains, i.e., one Gram-positive bacterium, *Staphylococcus aureus*, and two Gram-negative bacteria, *Escherichia coli* and *Proteus vulgaris*, were obtained from the National Public Health Laboratory (NPHL), Kathmandu, and growth conditions were maintained according to the method described previously [3] with some modifications.

### 3.2. Well Diffusion Method

The well diffusion method was done to determine the antibacterial activity of plant extract according to the method described previously [13] with some modifications. Wells (8 mm diameter and about 2 cm apart) were made in each of the plates using a sterile cork borer. A stock solution of each plant extract was prepared at a concentration of 1 mg/ml and 2 mg/ml in 10% dimethyl sulfoxide (DMSO) solution. A volume of 100 *μ*l of stock solution of plant extract was added by sterile syringe into the wells and allowed to diffuse at room temperature for 2 hr. Ofloxacin and cefpodoxime were taken as positive control where 10% DMSO solution was taken as a negative control. Then, medium plates were incubated at 37°C for 24 hr in an incubator. After 24 hr, zones of inhibition were measured.

### 3.3. Antioxidant Activity Assay

The antioxidant activity of the plant extract was determined using DPPH radical scavenging assay and nitric oxide (NO) radical scavenging assay methods.

### 3.4. DPPH Radical Scavenging Assay

DPPH is a free radical-generating compound which is used to determine the radical scavenging activity of extracts. It is a rapid, simple, and inexpensive method to measure antioxidant capacity. The odd electron in the DPPH free radical gives a strong absorption maximum at 517 nm and has a deep purple color [5].

DPPH free radical assay was performed according to the method described previously [14] with some modifications. In brief, 2 ml of different extract solution was mixed with 2 ml of DPPH solution (60 *μ*M) and allowed to stand for 30 min. Then, absorbance of each plant samples was measured at 517 nm by using a UV spectrophotometer. Radical scavenging activity of each sample was calculated by using the following formula:

(1) $$\begin{array}{c} \text{radical scavenging activity }\left(\%\right)=\left[\frac{\left(A_{0}-A_{s}\right)}{A_{0}}\right]\times 100\%, \end{array}$$

where *A*_*0*_ = absorbance of control and *A*_*S*_ = absorbance of a sample. Control is the test solution without a sample. A similar process was done with an ascorbic acid solution of concentrations (100 *μ*g/ml, 10 *μ*g/ml, and 1 *μ*g/ml). Ascorbic acid was taken as a positive control.

### 3.5. NO Radical Scavenging Assay

#### 3.5.1. Preparation of Griess Reagent

A total amount of 0.25 g of NEDD was mixed in sufficient deionized water to produce 250 ml of 0.10% of the NEDD solution. Then, 1% sulfanilamide solution was prepared by dissolving 2.50 g of sulfanilamide in 5% phosphoric acid to produce 250 ml. Finally, 500 ml of Griess reagent was prepared by mixing NEDD solution and sulfanilamide solution which was then stored in a refrigerator and used before 8 hr.

#### 3.5.2. Determination of NO Radical Scavenging Assay

In NO scavenging assay, incubation of solutions of sodium nitroprusside in phosphate buffer saline at 250°C for 2.5 hr resulted in a linear time-dependent nitrite production [15]. The quantities of NO produced is determined using Griess reagent by the development of purple to pink color during the diazotization of nitrite with sulfanilamide and its subsequent coupling with NEDD. The absorbance is observed at 548 nm on UV spectrophotometer [16].

NO radical scavenging activity was measured by the method described previously [1] with slight modifications. In brief, 1 ml of test samples of different concentrations were taken in a test tube and mixed with 1 ml of sodium nitroprusside (5 mM) solution and test tubes were incubated for 2.5 hr at 29°C. After 2.5 hr, 2 ml Griess reagent was added to each test tubes and absorbance was measured at 548 nm in a UV spectrophotometer after 30 min incubation at room temperature. The radical scavenging effects of test samples were calculated by the following formula:

(2) $$\begin{array}{c} \text{radical scavening activity}\left(\%\right)=\left[\frac{A_{0}-A_{s}}{A_{0}}\right]\times 100\%, \end{array}$$

where *A*_*o*_ = absorbance of control and *A*_*s*_ = absorbance of sample.

Control is the test solution without the test sample. A similar process was done for curcumin solution used as positive control, i.e., standard solution.

## 4. Results

### 4.1. Extraction Yield Value

The extract yield % was calculated using equation (3). The extract yield % of the plant sample is shown in Table 1. The extract yield percentage was relatively higher in root and leaves of *M. rubicaulis*, and flower of *R. indica* while relatively lower in the stem of *M. rubicaulis* and leaves of *R. indica*.

(3) $$\begin{array}{c} \%\text{yield}=\frac{\text{weight of extract yield}}{\text{weight of crude sample taken}}\times 100\%. \end{array}$$

### 4.2. Antibacterial Activity

Each of different parts of plant extract, i.e., root, stem, and leaves of *M. rubicaulis*, and flower and leaves of *R. indica* were used to measure and compare the zone of inhibitions with the standard antibiotics ofloxacin and cefpodoxime at a concentration of 1 mg/ml and 2 mg/ml in 10% DMSO in test microorganisms, i.e., two Gram-negative bacteria, which are *E. coli* and *P. vulgaris*, and one Gram-positive bacteria, which are *S. aureus*. The standard drug, ofloxacin had shown zone of inhibition towards *E. coli* (45 mm), *P. vulgaris* (48 mm), and S. *aureus* (34 mm), respectively. Similarly, cefpodoxime had shown zone of inhibition towards *E. coli* (36 mm), *P. vulgaris* (34 mm), and S. *aureus* (24 mm), respectively. The root extract of *M. rubicaulis* was found to have a weak zone of inhibition towards *S. aureus*, i.e., 12 mm, and leaves and stem extracts were found to have a weak zone of inhibition towards *P. vulgaris*, i.e., 11 mm and 11 mm, respectively. However, the flower and leaves extract of *R. indica* were found to have no zone of inhibition towards the selected bacteria even in 2 mg/ml of concentration. At a concentration of 1 mg/ml, none of the extracts had shown a zone of inhibition towards the selected organism.

### 4.3. Antioxidant Activity

#### 4.3.1. DPPH Radical Scavenging Activity

**Figure 1:**
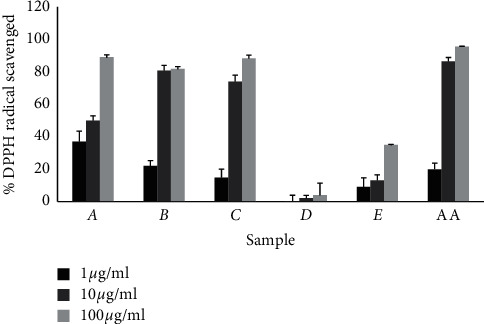
Graphical representation of percentage scavenging of DPPH free radicals by extract and ascorbic acid (AA) at 517 nm. The error bar represents the standard deviation of three independent determinations performed in triplicate of sample size (*n* = 6).

The result of the DPPH radical scavenging assay is shown in Table 2 and Figure 1. Among the studied plant extracts, root, leaves, and stem extracts of *M. rubicaulis* exhibited high antioxidant activity in DPPH free radical scavenging assay as 89%, 88%, and, 81%, respectively, in 100 *µ*g/ml. The half maximal inhibitory concentration (IC_50_) value of leaves extract was 9.80 *µ*g/ml. Similarly, the IC_50_ value of root extract and stem extract was found to be 10.19 *µ*g/ml and 13.64 *µ*g/ml, respectively, which was very close to that of standard (ascorbic acid) as 9.99 *µ*g/ml. The leaves extract of *R. indica* exhibited DPPH radical scavenging assay as 34% with an IC_50_ value of 19.73 *µ*g/ml, but flower extract of *R. indica* exhibited low antioxidant activity, i.e., 4% with an IC_50_ value of 165.36 *µ*g/ml. IC_50_ is defined as the amount of antioxidant required to inhibit 50% of DPPH free radicals under the experimental conditions.

### 4.4. NO Scavenging Activity

**Figure 2:**
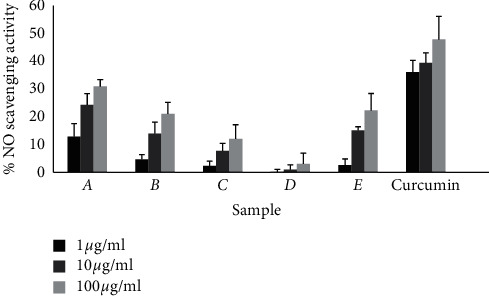
Graphical representation of percentage scavenging of NO free radicals by extract and curcumin at 548 nm. The error bar represents the standard deviation of three independent determinations performed in triplicate of sample size (*n* = 6).

The NO scavenging activity of different plant extracts was determined by using Griess reagent. Sodium nitroprusside was used as nitric oxide-producing agent. The root and stem of *M. rubicaulis* and leaves of *R. indica* had shown NO scavenging activity at higher concentrations (100 *µ*g/ml), i.e., 31% and 21% and 22%, respectively. The root, stem, and leaves extracts of *M. rubicaulis* had shown IC_50_ value as slightly higher than that of standard drug curcumin as 0.23 *µ*g/ml, 0.32 *µ*g/ml, and 0.58 *µ*g/ml, respectively. Similarly, the extract of *R. indica* had also exhibited higher IC_50_ value to that of standard, which is shown in Figure 2 and Table 2.

The result of the IC_50_ value of samples from DPPH scavenging assay against the DPPH free radical and NO scavenging assay against the nitric oxide free radical is in Table 2. Ascorbic acid had shown a good IC_50_ value of 9.99 *µ*g/ml against DPPH free radical, but curcumin had exhibited an IC_50_ value of 0.12 *µ*g/ml towards nitric oxide free radical. However, both the extracts of plant samples had shown IC_50_ value nearly similar to that of standard as ascorbic acid and curcumin which is shown in Table 2.

## 5. Discussion

Antibacterial activities were assayed to determine the zone of inhibition towards *E. coli*, *P. vulgaris*, and *S. aureus* at a concentration of 1 mg/ml and 2 mg/ml. The root, stem, and leaves extract of *M. rubicaulis* were found to have a negligible zone of inhibition, but flower and leaves of *R. indica* were found to have no zone of inhibition as compared to the reference drug ofloxacin and cefpodoxime even at a concentration of 2 mg/ml, but no zone of inhibition was shown by any selected plants extract at 1 mg/ml which is shown in Table 2. This activity was comparatively lower than that observed in methanolic leaves extract of *M. rubicaulis* by the agar diffusion method against Gram-negative (*E. coli*), at a concentration of 15 mg/ml [17] and methanolic root extract of *M. rubicaulis* at a concentration of 1000 mcg/ml (*E. coli*) by the cup plate method [18]. No zones of inhibition were seen for both ethanolic leaves and root extracts of *M. rubicaulis* at 2 mg/ml by using a well diffusion method towards *E. coli.* It may be due to the solvent effect as well as concentration effect.

The antioxidant activities were assayed by DPPH free radical scavenging and NO scavenging assays. The DPPH free radical is a simple and acceptable method to evaluate the antioxidant activity of plant extracts. The DPPH forms a stable molecule on accepting an electron or a hydrogen atom and thus has applications in the determination of radical scavenging activity of natural products as well as synthetic compounds [19]. The leaves, root, and stem extracts of *M. rubicaulis* exhibited high antioxidant activity by DPPH free radical scavenging assay with an IC_50_ value of 9.80 *µ*g/ml, 10.19 *µ*g/ml, and 13.64 *µ*g/ml, respectively, which was very close to that of standard (ascorbic acid) shown in Table 2 and Figure 1. It may suggest that the extract might contain an electron donor molecule that reacts with free radicals to convert them to more stable products and terminate radical chain [20].

NO is an important chemical mediator involved in the regulation of various physiological processes. NO or RNS (reactive nitrogen species) formed during their reaction with oxygen or with superoxides, such as NO_2_, N_2_O_4_, and N_3_O_4_ which are very reactive and are responsible for altering the structural and functional behavior of many cellular components [21]. Scavengers of nitric oxide compete with oxygen leading to reduced production of nitrite ions [22]. Among the plant extract under study, the root extract of *M. rubicaulis* showed the NO scavenging activity by 31% at 100 *µ*g/ml and other extracts showed less activity. The IC_50_ value of both the plant samples had shown similar value to that of the standard drug, curcumin, which is shown in Table 2. The ability of the extract to generate the nitrite in decreased amount may be due to the capacity to decompose the sodium nitroprusside in vitro due to the presence of different phytoconstituents mainly phenolic compounds such as flavonoids responsible to scavenge the free radical by competing with the oxygen molecule of NO free radical, thus leading to antioxidant activity [16]. This activity was comparatively lower than that observed in water and carbinol extract of leaves of *R. indica* in previous studies [1] whereas ethanolic leaves extract of *R. indica* showed less activity in this study. This may be due to the solvent effect.

## 6. Conclusion

Out of two plant species, *M. rubicaulis* had shown antibacterial activity towards *P. vulgaris* and *S. aureus*, but antioxidant activity was shown by both plant species. Antibacterial and antioxidant activities found in plant species might help in formulation development which could be marketed as antibacterial and wound-healing agents in future days.

## Figures and Tables

**Table 1:** Extraction yield value and symbol code used for plant samples.

Scientific name	Parts used	Sample code	Percentage yield value (ethanol)
*Mimosa rubicaulis*	Root	A	8
	Stem	B	3.2
	Leaves	C	11.52

*Reinwardtia indica*	Flower	D	7.95
	Leaves	E	3.52

**Table 2:** Result of IC_50_ value of samples and standard drugs from DPPH scavenging activity and NO scavenging activity.

Samples	DPPH scavenging activity IC_50_ (*µ*g/ml)	NO scavenging activity IC_50_ (*µ*g/ml)
A	10.19	0.23
B	13.64	0.32
C	9.80	0.58
D	165.36	1.82
E	19.73	0.28
AA	9.99	—
Curcumin	—	0.12

## Data Availability

The data will be available upon request to the corresponding author.
